# Breastfeeding and the Risk of Illness among Young Children in Rural China

**DOI:** 10.3390/ijerph16010136

**Published:** 2019-01-07

**Authors:** Shanshan Li, Ai Yue, Cody Abbey, Alexis Medina, Yaojiang Shi

**Affiliations:** 1 Center for Experimental Economics in Education (CEEE), Shaanxi Normal University, Xi’an 710119, China; lishanshan.ceee@gmail.com (S.L.); shiyaojiang7@gmail.com (Y.S.); 2 Rural Education Action Program, Freeman Spogli Institute for International Studies, Stanford University, Palo Alto, CA 94305, USA; cjabbey88@gmail.com (C.A.); amedina5@stanford.edu (A.M.)

**Keywords:** breastfeeding, infants, illness, rural China

## Abstract

Poor rural areas in China exhibit the country’s highest rates of child mortality, often stemming from preventable health conditions such as diarrhea and respiratory infection. In this study, we investigate the association between breastfeeding and disease among children aged 6–24 months in poor rural counties in China. To do this, we conducted a longitudinal, quantitative analysis of socioeconomic demographics, health outcomes, and breastfeeding practices for 1802 child–caregiver dyads across 11 nationally designated poverty counties in southern Shaanxi Province in 2013–2014. We found low rates of continued breastfeeding that decreased as children developed: from 58.2% at 6–12 months, to 21.6% at 12–18 months, and finally to 5.2% at 18–24 months. These suboptimal rates are lower than all but one other country in the Asia-Pacific region. We further found that only 18.3% of children 6–12 months old met the World Health Organization (WHO)-recommended threshold for minimum dietary diversity, defined as consuming four or more of seven specific food groups. Breastfeeding was strongly associated with lower rates of both diarrhea and cough in bivariate and multivariate analyses. As the first analysis to use longitudinal data to examine the relationship between continued breastfeeding and child illness in China, our study confirms the need for programmatic interventions that promote continued breastfeeding in order to improve toddler health in the region.

## 1. Introduction

Breastfeeding is widely recognized as the best feeding practice for early child development [1]. During the first two years of a child’s life, breastfeeding can positively contribute to both short- and long-term health outcomes [2,3]. According to the World Health Organization (WHO), exclusive breastfeeding reduces infant mortality due to common childhood diseases such as diarrhea or pneumonia, both by increasing resistance to and allowing for a quicker recovery from such diseases. For children aged 6 to 23 months, continued breastfeeding reduces their exposure to contaminated food and liquids, and reduces the prevalence of diarrhea and cough [4,5]. Breastfeeding has also been associated with lower rates of other common childhood diseases, including upper respiratory tract illnesses [6]. Based on such evidence, the WHO recommends that children be exclusively breastfed for the first six months after birth, with breastfeeding continuing up to 2 years of age (or even later) together with the introduction of appropriate complementary foods [7].

A considerable amount of literature shows that rates of breastfeeding in rural China are dismally low, both in terms of exclusive breastfeeding in the first 6 months (21% to 29%) [8,9,10,11], and in terms of continued breastfeeding, defined as breastfeeding from 6–24 months of age. The rate of continued breastfeeding is 29.7% at 12 months, 7.9% at 18 months, and only 2.3% at 24 months [12]. A study conducted in 26 poor rural counties in 12 central and western provinces of China found that while just over half (55.5%) of children had continued breastfeeding for 1 year, only 9.4% of children had continued breastfeeding for 2 years [9]. These proportions lag behind the overall rates of continued breastfeeding in developing countries, which are 86% for children aged 6–11 months, and 68% for children aged 12–23 months [13]. 

In addition to encouraging continued breastfeeding, the WHO guidelines also indicate that complementary foods should be gradually introduced, beginning at around 6 months of age, and that by 1 year of age they should constitute the majority of a child’s diet [7,14]. Many studies have noted the important role that complementary foods play in shaping children’s health and nutrition [15,16]. Evidence suggests that higher dietary diversity may reflect better diets and diets that are more likely to meet daily energy and nutrient requirements [17,18]. In one study in Bangladesh, when continued breastfeeding was accompanied with complementary foods, there was a reduction in clinical malnutrition [19]. However, other evidence has shown either no link between continued breastfeeding and malnutrition [20], or that children with continued breastfeeding were less likely to consume more food items, suggesting a negative link between breastfeeding and dietary diversity [21]. Beyond a single, very small, geographically limited study [22], little is known about dietary diversity among young children in rural China.

Concurrent with the low rates of breastfeeding are studies showing the worrisome status of child health in China, especially in rural areas. Although evidence has shown that China has achieved a rapid reduction in child mortality in recent decades, this masks great disparities across regions [23]. Children living in poor rural areas have significantly worse health outcomes than their wealthier, urban counterparts [24,25]. Evidence shows that more than half (69.5%) of rural children under 2 years old suffer from diarrhea [26]. This proportion is higher than that observed in developing countries (40.3–51%) [27,28]. The prevalence of fever and cough among children aged 6–11 months is also high in rural China, at 49.7% and 59.5%, respectively [22]. These rates are not only higher than those observed in urban areas in China (12.2% for fever, 19.9% for cough) [5,29], but also higher than in some developing countries (11–19.5% for cough; 12–51% for fever) [5,30]. This is especially alarming, considering that China is now officially classified as a middle-income country [31].

Could the low rates of continued breastfeeding be connected with the high prevalence of poor child health outcomes in China? While several studies (mentioned above) have measured the prevalence of breastfeeding among various populations within China [9,10,11,12], to our knowledge, existing studies have yet to explore the relationship between the low rate of continued breastfeeding and child health outcomes, especially in poor rural areas. 

The overall objective of this paper is to describe the status quo of child health status and early childhood feeding behaviors in our sample and investigate the association between continued breastfeeding and the incidence of child health outcomes (i.e., fever, diarrhea, upper respiratory infection, and cough) among children aged 6–24 months in poor, rural counties in China. 

## 2. Materials and Methods 

### 2.1. Sample Selection 

Our study was conducted in 2013 in 11 nationally designated poverty counties located in southern Shaanxi Province, China. The area is predominantly Han Chinese and had a per capita annual income of about 6502 RMB (1050 USD) in 2013 [32], over 25% lower than the 8896 RMB (1437 USD) national rural average in the same year [33]. From each of these 11 counties, all townships (the middle level of administration between county and village) were selected to participate in the study. There were two exceptions to this rule: we excluded the one township in each county that housed the county seat, and we excluded any townships that did not have any villages with a population of 800 or more. In total, according to these criteria, 174 townships were included in the study.

The sample villages were originally selected as follows. To meet the power requirements of a larger, interventional study (not reported in this paper) [34], we required a minimum of five children in each township. With this requirement in mind, we first randomly selected one village (with a population of 800 or more) from each township to participate. A list of all registered births over the past 12 months was obtained from the local family planning official in each village. All children in our desired age range (6–12 months) were enrolled in the study. If a village had fewer than five children in our desired age range, we randomly selected an additional village in the same township for inclusion in the study and continued to randomly select additional villages until five children per township had been found.

In total, we included 1802 children aged 6–12 months at the time of the baseline survey. We conducted two additional rounds of follow-up data collection. The children were 12–18 months old during the first follow-up survey, and 18–24 months old during the second follow-up survey. 

### 2.2. Data Collection

Teams of trained enumerators collected socioeconomic information from all households participating in the study. We collected both household-level and child-specific data. At the household level, the primary caregiver (typically either the child’s mother or grandmother) was identified in each family as the individual who was most responsible for the child’s care. She (or he) was administered a detailed survey on household characteristics, including maternal age and education, and whether the family was receiving Minimum Living Standard Guarantee Payments (a form of government welfare for the lowest-income families nationwide). She also provided information on household assets, which was used to calculate the household socioeconomic status (SES) index using principal component analysis [35]. Questions included whether or not the household owned or had access to tap water, a heater, a refrigerator, a washing machine, a computer, internet, a motorcycle, a car, a truck, a flushing toilet, and the approximate housing value. At the individual level, the primary caregiver also provided information on each child’s gender and birth order. The child’s age was obtained from his or her birth certificate. 

The survey also included a detailed module on feeding practices, based on the “Indicators assessing children and young child feeding practices” [36,37] complied by the WHO and others in the international community. We asked caregivers whether the child was still being breastfed at the time of survey administration, as well as whether they were being fed formula. The main predictor was the status of continued breastfeeding, which was defined as whether the child was still being breastfed at the time of each survey round. A child was considered to be exclusively breastfed if he or she was fed nothing but breast milk on the day prior to survey administration. We also provided caregivers with a list of seven different food categories: 1. grains, roots, and tubers; 2. legumes and nuts; 3. dairy products; 4. flesh foods (meat, fish, poultry, and liver/organ meats) 5. eggs; 6. vitamin-A-rich fruits and vegetables; 7. other fruits and vegetables. We then asked caregivers to identify which of the seven food types their child had consumed the previous day. Following WHO guidelines [38], a child was defined as having met the minimum dietary diversity requirements if they had consumed foods from four or more different categories.

The primary outcomes of interest for this study were incidence of fever, diarrhea, cold, and cough among sample children. These were collected through caregiver recall of symptoms in the month prior to survey administration. All variables used in our statistical analysis are presented in Appendix Table A1.

### 2.3. Ethical Approval

Ethical approval was provided by the Institutional Review Board (IRB) of Stanford University (Protocol ID 25734) and from the Ethical Review Board of Sichuan University (Protocol ID 2013005-01). All participants gave oral consent for both their own and their children’s involvement in the study.

### 2.4. Statistical Analysis

Descriptive statistics, including patterns of breastfeeding and basic background characteristics, were reported. Multivariate regressions with random effects at the household level were used to control for intra-household correlation across survey waves. Building on the related literature [39,40], we considered the household level, child-specific characteristics, and child’s dietary diversity described above as potential confounders of association between breastfeeding and child illness. Confounding factors were controlled for in the final model. In estimating the correlation between continued breastfeeding and health problems, we used ordinary least squares (OLS), including a set of covariates in a regression on the health status of children. We first ran an unadjusted regression (1):

*Y_ij_ = α + β ∗ BFS_ij_ + ε_ij_* (1)

The dependent variable *Y_ij_* indicates the rate of diarrhea/cough/fever/cold of children *i* in village *j,* which equals to 1 if the child had experienced diarrhea/cough/fever/cold in the month prior to survey administration. *BFS_i_* indicates whether the child was still being breastfed at the time of each survey round, which indicates the caregiver feeding practice for child *i.* Standard errors are clustered at the village level. *ε_ij_* is an error term.

To control for the potential confounding effects of child and family characteristics, we ran a multivariate analysis building on Equation (1) above by including a vector of control variables.

*Y_ij_ = α + β ∗ BFS_ij_ + γX_ij_ + ε_ij_* (2)

The term $X_{ij}$ is a vector of covariates including the child’s dietary diversity, child characteristics (age, gender, sibling, etc.) and household characteristics (maternal education level, maternal age, etc.). 

In all Equations (1)–(2), we computed heteroskedasticity-robust standard errors (adjusted for clustering at village level).

All statistical analyses were carried out using Stata 14.2. *p*-values below 0.05 were considered statistically significant [9]. 

## 3. Results

### 3.1. Attrition Across Survey Waves

The baseline sample included 1802 children. We were able to follow up with 1592 children after 6 months (first follow-up, when children were aged 12–18 months) and with 1499 children after 12 months (second follow-up, when children were aged 18–24 months). The attrition of the sample was therefore 11.7% after 6 months, and 16.8% after 12 months. Attrition was random and not associated with variables of interest. Overall, attrition was low between the time of the baseline to the final survey, and not correlated with any key outcome variables (Appendix Table A2).

### 3.2. Socioeconomic and Demographic Characteristics of Participants

The basic socioeconomic and demographic characteristics of study participants are reported in Table 1. Children were aged 6–12 months at the time of the baseline survey, and the sample was evenly distributed across ages (data not shown). The vast majority of children in our sample (79.3%) did not have siblings at baseline. The mother was the primary caregiver for 82.4% of the children in the sample when the children were 6–12 months old, though this percentage decreased to 63.0% when the children were 18–24 months old, as mothers out-migrated (data not shown). The majority of sample mothers (78.7%) had completed 9 years of schooling or less, and around half (50.1%) were over 25 years of age. About one quarter (23.6%) of sampled families reported receiving Minimum Living Standard Guarantee payments.

### 3.3. Feeding Behaviors of Children in Rural China

In Table 2, we show that fewer than 1% of children were being exclusively breastfed at the time of the baseline survey, when they were 6–12 months old (0.33%). No children older than 12 months were exclusively breastfed. The rate of any breastfeeding was low across all age groups, and showed a decreasing trend as children aged, with the highest rate at baseline (58.2%), decreasing to 21.6% when children were 12–18 months old, and further decreasing to 5.2% when the children were 18–24 months of age. As the sample of children who were still being breastfed after 18 months was too small to be meaningful (5.2%), we did not include data from the second follow-up survey in any of our remaining analyses (when children were 18–24 months old).

### 3.4. Dietary Diversity in Children Aged 6–18 Months in Rural China

Table 3 shows the dietary diversity of children in our sample. Overall, children aged 6–12 months only consumed an average of 3.0 types of food on the day prior to survey administration. This is significantly lower than the recommended minimum diversity threshold set by WHO, which is defined as four or more of the seven food groups [38]. Only 18.3% of children aged 6–12 months met the minimum dietary diversity threshold. By the time the children reached 12–18 months of age, however, this ratio had increased substantially to 71.8%. We found that children who were breastfed had significantly less diverse diets than children who were not breastfed (including both those who had formula with food and those who ate food alone). This was true across both age groups. From this table, it can also be seen that the composition of the children’s diets became more diverse as they grew older, from 6–12 months to 12–18 months. 

### 3.5. Health Status of Children in Rural China

Table 4 summarizes the incidence of fever, diarrhea, cold, and cough among sample children, showing that the incidence of illness decreased as children aged. Incidence of diarrhea decreased from 36.9% when the children were 6–12 months old, to 30.4% when they were 12–18 months old. Similarly, the incidence of cough decreased from 45.5% to 42.0% across the same time period. The incidence of fever decreased from 30.0% to 28.5%, and the incidence of cold decreased from 56.9% to 54.8%.

### 3.6. Links between Feeding Behavior and Health Status of Children

Next, we investigated whether there was a correlation between feeding behavior and child health status. We found that breastfed children had lower rates of both diarrhea and cough, both at 6–12 months and at 12–18 months (Table 4). However, we found no differences in incidence of fever or cold. 

In order to further examine the relationship between feeding behavior and the health status of children, we ran a series of multivariate regressions controlling for children, maternal, and household characteristics, presented in Table 5. We found that the multivariate analysis results were consistent with the findings of the bivariate analysis; that is, children who were breastfed between 6 and 18 months had lower incidence of both diarrhea and cough. Specifically, in households with continued breastfeeding, the incidence of diarrhea was 11.9% lower than that of weaned children, while the incidence of cough was 7.0% lower. However, as in the bivariate analysis, there was no significant correlation between continued breastfeeding and incidence of either fever or cold.

When comparing the association between breastfeeding and rate of illness across the age group (Table 6), we found that the results were consistent across both age groups. The rate of diarrhea was significantly lower for breastfed children at both 6–12 months and 12–18 months of age when compared to non-breastfed children. Breastfed children also had significantly lower rates of cough at 12–18 months (though not at 6–12 months). In addition, across both age groups, breastfeeding was not significantly correlated with fever or cold.

## 4. Discussion

This study shows that the practice of continued breastfeeding for children between 6 and 24 months in rural China is suboptimal. The rate of continued breastfeeding for children aged 6 to 12 months old was only 58.2%, and declined to 5.2% for children aged 18 to 24 months. WHO guidelines clearly state that both of these rates should be 100%. These rates of continued breastfeeding are lower than almost all other countries in the Asia-Pacific region, with only one other country—Thailand—reporting a lower rate of continued breastfeeding at one year of age (32%) [41]. 

Our study also sheds light on the degree of dietary diversity among children in rural China, suggesting that their diets may be insufficiently diverse. Based on available evidence, complementary foods should be introduced to children at 6 months of age, as dietary diversity is strongly and consistently associated with all measures of nutritional status [42]. In our data, however, we find that the average number of food groups consumed is 2.5 for children aged 6–12 months and 3.2 for children aged 12–18 months—significantly lower than the minimum threshold set by the WHO. Interestingly, children who were not breastfed had higher dietary diversity than those who were (a phenomenon which has been documented by previous research [42]), though the level of dietary diversity was still insufficient regardless of breastfeeding status. This result may stem from a variety of different reasons. One important factor may be lack of access to information about child feeding practices. One qualitative study of caregiver feeding practices in rural China found that many rural caregivers lacked information on best feeding practices for their young children, and that they largely believed that as long as a child was not hungry, he or she was getting adequate nutritional intake [43]. If children who are breastfed feel fuller longer, then their caregivers may feel less urgency in introducing more and more varied complementary foods. Household income may also be a factor. Since breastfeeding is sometimes seen as a low-cost alternative to formula feeding, it may be that the poorer families are engaging in continued breastfeeding, and are therefore also less likely to have high levels of dietary diversity due to income constraints. A related issue may be that of migration. If a mother out-migrates from home for employment opportunities, she is substituting a higher household income (via remittances) for lost breastfeeding opportunities.

We note that the WHO urges researchers to use caution in comparing dietary diversity between breastfed and non-breastfed samples, due to the possibility that non-breastfed children may be consuming higher levels of animal milk as a substitute for breast milk. In our sample, however, we found no difference in dairy consumption between breastfed and non-breastfed children (data not shown), and therefore have no reason to believe that differences in dairy consumption may be influencing our results.

Despite lower dietary diversity, the breastfed children in our sample were less likely to contract illnesses, thereby compensating—at least in part—for the lack of diet diversity. The continued breastfeeding of children aged 6 to 18 months was linked with significantly lower incidence of both diarrhea and cough. This finding confirms the benefits of continued breastfeeding for children aged between one and two years old, as suggested by the United Nations International Children’s Emergency Fund (UNICEF) [44].

Interestingly, we did not find that continued breastfeeding was associated with the incidence of fever or cold. Studies elsewhere have found that breastfeeding may be associated with a lower risk of fever in specific cases, including post-immunization, as well as among children with hand, foot, and mouth disease [40,45]. However, we do not know the specific underlying causes of fever in our data. Given that the cause of fever is highly variable, and its relation with breastfeeding is therefore complicated, we cannot identify the reasons for why there were no such correlations in our data. As for the correlation between breastfeeding and cold, one possible reason may be that children in rural areas are exposed to more infectious organisms that may reduce the protective effect of breast milk to a certain extent. 

To our knowledge, ours is the first study using longitudinal data to examine the relationship between continued breastfeeding and child illness in poor rural counties in China. It was based on a large sample of children, representative of poor counties in northern China. 

One limitation of this study is that because our data on child feeding practices is based on caregiver recall, we cannot rule out the possibility of recall bias. Also, although we attempted to sample villages that varied in terms of household income, population size, distance from county seat, and geographic location, it is possible that the sample is not representative of all households throughout all of rural China.

In conclusion, we find that continued breastfeeding after 6 months can help to provide protection against diarrhea and cough. We also find, however, that the practice of continued breastfeeding in rural China is uncommon, especially after one year of age. A qualitative study conducted in the southern part of Shaanxi Province suggests that a lack of information on healthy feeding practices and a poor understanding of the nutritional contents of food may be behind these suboptimal feeding practices [43]. Our results indicate that efforts should be made to improve the quality of information available to caregivers of young children and increase the rate of continued breastfeeding in both rural China and other low-resource settings in order to improve child health. One method of doing so might be to launch a series of public health and nutrition campaigns in rural areas, as such efforts have been shown to improve caregivers’ feeding practices [46]. Community health worker programs have also been effective in other contexts [47,48,49], and some evidence suggests that such an approach may show promise in rural China as well [50]. Any campaign should provide specific child-feeding information, such as the importance of continued breastfeeding and dietary diversity for children’s health and development, and how to provide children with a rich diet.

## Figures and Tables

**Table 1:** Summary statistics of sample children aged 6–12 months in rural China (*n* = 1802).

	Frequency (*n*)	Percentage (%)
Panel A: Child Characteristics		
(1) Male	949	52.7
(2) Has Siblings	373	20.7
(3) Premature Birth	198	11.0
(4) Mother is the Primary Caregiver	1477	82.4
Panel B: Household Characteristics		
(5) Maternal Age >25	903	50.1
(6) Years of Maternal Education		
≤6 years	497	27.6
>6 and ≤9 years	1008	55.9
>9 years	297	16.5
(7) Years of Paternal Education		
≤6 years	413	22.9
>6 and ≤9 years	1005	55.8
>9 years	384	21.3
(8) Father at Home	790	43.8
(9) Grandmother is Healthy	761	42.2
(10) Years of Grandmother Education		
≤6 years	1491	82.7
>6 and ≤9 years	263	14.6
>9 years	48	2.7
(11) Asset Index		
Poorest tercile Middle tercile Wealthiest tercile	600 601 601	−1.2 ± 0.4 −0.2 ± 0.3 1.4 ± 0.7
(12) Family Receives Minimum Living Standard Guarantee Payments	425	23.6

Notes: These are the descriptive statistics of child and household characteristics for infants aged 6–12 months. The table shows the mean and standard deviation of infant and household characteristics for the full sample. The variable “Father at Home” equals 1 if, at the time of the baseline survey, the father had been at home for a majority of the previous six months, and 0 otherwise. “Grandmother Healthy” is based on self-reported general health. The asset index was constructed using polychoric principal components on the following variables: tap water, toilet, water heater, washing machine, computer, fridge, air conditioning, motor or electronic bicycle, and car.

**Table 2:** Feeding behaviors among sample children aged 6–24 months in rural China (*n* = 4893).

	6–12 Months	12–18 Months	18–24 Months
	Number (%)	Number (%)	Number (%)
	(1)	(2)	(3)
(1) Any Breastfeeding	1049 (58.2)	343 (21.5)	78 (5.2)
(1.1) Exclusive Breastfeeding	6 (0.3)	0 (0.0)	0 (0.0)
(1.2) Continued breastfeeding, without Formula	779 (43.2)	240 (15.1)	49 (3.3)
(1.3) Continued breastfeeding, with Formula/Food	264 (14.7)	103 (6.4)	29 (1.9)
(2) Any Formula-fed			
(2.1) Formula with Food	733 (40.7)	1033 (64.9)	1086 (72.5)
(3) Food Alone	20 (1.1)	216 (13.6)	335 (22.4)
(4) Total	1802	1592	1499

Notes: There are three main types of feeding behaviors among the sample (“Any Breastfeeding”, “Any Formula-fed” and “Food Alone”). “Any Breastfeeding” includes all sample children who are consuming breast milk. “Exclusive Breastfeeding” refers to children who consume nothing but breast milk. Sample children who were fed breast milk and food (but no formula) were classified as “Non-Exclusive Breastfeeding without Formula”. Sample children who were fed breast milk, food, and formula were classified as “Non-Exclusive Breastfeeding with Formula”. No sample children were fed breast milk and formula but no food. “Any Formula-fed” refers to sample children who were fed formula with food. No sample children were fed formula alone. All children in our desired age range (6–12 months) were enrolled in baseline survey. This was defined so as to include children who were as young as 6 months and zero days (day of their 6-month birthday), as well as children who were as old as 12 months and 30 days (one day before their 13-month birthday). This means that the sample children were 12–18 months old (12 months and zero days, and 18 months and 30 days) during the first follow-up survey (6 months after the baseline survey), and 18–24 months old during the second follow-up survey (12 months after the baseline survey).

**Table 3:** Descriptive statistics of dietary diversity in infants aged 6–18 months in rural China (*n* = 3394).

	6–12 Months		12–18 Months	
	Percent/Mean	*n*	Percent/Mean	*n*
Percentage of children who met the minimum dietary diversity threshold	18.3	330	71.8	1143
Mean number of food types child consumed yesterday				
(1) Full sample	3.0	1802	4.2	1592
(2) Breastfeeding sample	2.8	1049	3.7	343
(3) Non-Breastfeeding sample	3.3	753	4.4	1249
(3.1) Formula with food	3.3	733	4.5	1033
(3.2) Food alone	3.0	20	4.1	216
*p*-values				
(1) Difference (2)–(3)	<0.01		<0.01	
(2) Difference (2)–(3.1)	<0.01		<0.01	
(6) Difference (2)–(3.2)	0.35		<0.01	

Notes: The unit for the dietary diversity data is the mean number of the following food categories fed to the child on the day prior to survey administration: 1. grains, roots, and tubers; 2. legumes and nuts; 3. dairy products; 4. flesh foods (meat, fish, poultry, and liver/organ meats) 5. eggs; 6. vitamin-A-rich fruits and vegetables; 7. other fruits and vegetables. There is no “formula alone” or “formula with breastfeeding but without food”.

**Table 4:** Correlation between breastfeeding and health outcomes in children aged 6–18 months in rural China (*n* = 3394).

	6–12 Months			Difference *p*-Value	12–18 Months			Difference *p*-Value
	Total	Breastfeeding			Total	Breastfeeding		
		Yes	No			Yes	No	
	(1)	(2)	(3)	(2)–(3)	(4)	(5)	(6)	(5)–(6)
(1) Child had a fever in the past month	541 (30.0)	28.8(302)	31.7 (239)	0.18	428 (28.5)	27.1 (93)	26.8 (335)	0.53
(2) Child had diarrhea in the past month	664 (36.9)	33.2 (348)	42.0 (316)	<0.01	456 (30.4)	25.1 (86)	29.6 (370)	0.02
(3) Child had a cold in the past month	1025 (56.9)	57.9 (607)	55.5 (418)	0.32	823 (54.8)	54.2 (186)	51.0 (637)	0.82
(4) Child had a cough in the past month	819 (45.5)	43.3 (454)	48.5 (365)	0.03	631 (42.0)	34.4 (118)	41.1 (513)	<0.01
*n*	1802	1049	753		1592	343	1249	

Notes: Breastfeeding includes both exclusive breastfeeding and non-exclusive breastfeeding, with or without formula/food.

**Table 5:** Multivariate analysis: Correlations between breastfeeding and health outcomes in children aged 6–18 months in rural China.

		OLS	OLS
		(1)	(2)
(1) Child had a fever in the past month		−0.017	−0.014
		(0.018)	(0.021)
	*n*	3394	3394
(2) Child had diarrhea in the past month		−0.072 *	−0.119 **
		(0.017)	(0.020)
	*n*	3394	3394
(3) Child had a cold in the past month		0.027	0.004
		(0.021)	(0.024)
	*n*	3394	3394
(4) Child had a cough in the past month		−0.058 **	−0.070 **
		(0.021)	(0.024)
	*n*	3394	3394
(5) Dietary Diversity			Yes
(6) Controls			Yes
(7) Enumerator fixed effect			Yes
(8) Wave dummies		Yes	Yes

Notes: Column (1) shows the coefficients on breastfeeding in an ordinary least squares (OLS) regression pooling data across waves, controlling for survey wave dummies. Column (2) shows coefficients from pooled OLS regressions additionally controlling for the child’s dietary diversity and baseline controls (child age, gender, whether the child was premature, whether the child has siblings, maternal age, maternal educational level, paternal educational level, paternal migration status, health of the child’s grandmother, educational level of the child’s grandmother, asset index, and whether the household receives Minimum Living Standard Guarantee payments). Standard errors are clustered at the village level. *N* is the total number of observations in each regression. * indicates significance at 5%; ** indicates significance at 1% after adjusting for multiple hypotheses, using the step-down procedure of Romano and Wolf (2005) to control the familywise error rate (FWER).

**Table 6:** Multivariate analysis: Correlations between breastfeeding and health outcomes in infants aged 6–18 months in rural China.

	6–12 Months		12–18 Months	
	(1)	(2)	(3)	(4)
(1) Child had a fever in the past month	−0.030 (0.022)	−0.25 (0.029)	0.003 (0.026)	−0.003 (0.035)
(2) Child had diarrhea in the past month	−0.088 * (0.023)	−0.118 * (0.028)	−0.046 (0.026)	−0.118 * (0.034)
(3) Child had a cold in the past month	0.024 (0.025)	−0.003 (0.030)	0.032 (0.033)	0.013 (0.041)
(4) Child had a cough in the past month	−0.052 (0.028)	−0.052 (0.032)	−0.067 ** (0.031)	−0.094 ** (0.038)
(5) Dietary Diversity		Yes		Yes
(6) Controls		Yes		Yes
(7) Enumerator fixed effect		Yes		Yes
(8) Observations	1802	1802	1592	1592

Notes: Column (1) shows the coefficients on breastfeeding in an OLS regression pooling data across waves, controlling for survey wave dummies. Column (2) shows coefficients from pooled OLS regressions, additionally controlling for child’s dietary diversity and baseline controls (child age, gender, whether the child was premature, whether the child has siblings, maternal age, maternal educational level, paternal educational level, paternal migration status, health of the child’s grandmother, educational level of the child’s grandmother, asset index, and whether the household receives Minimum Living Standard Guarantee payments). Standard errors are clustered at the village level. * indicates significance at 1% after adjusting for multiple hypotheses using the step-down procedure of Romano and Wolf (2005) to control the familywise error rate (FWER); ** indicates significance at 5%.

## Appendix A

**Table A1:** Variables description.

Variables	Description
Panel A. Child Characteristics	
Gender (1 = Male, 0 = Female)	Child’s gender
Age (months)	Child’s age in months
Has siblings (1 = Yes, 0 = No)	Child has siblings from the same parents
Premature Birth (1 = Yes, 0 = No)	Child was born prior to 37 weeks of gestation
Mother is the primary caregiver (1 = Yes, 0 = No)	Mother is primarily responsible for the child’s care
Panel B. Household Characteristics	
Maternal Age >25 (1 = Yes, 0 = No)	Whether mother’s age is above or below 25 years
Maternal Education (1 ≥ 9 years, 0 ≤ 9 years)	Whether mother has received any high school education
Paternal Education (1 ≥ 9 years, 0 ≤ 9 years)	Whether father has received any high school education
Father lives at Home (1 = Yes, 0 = No)	Whether father lives at home with the child
Grandmother is Healthy (1 = Yes, 0 = No)	Health status of grandmother
Grandmother Education (1 ≥ 6 years, 0 ≥ 6 years)	Whether grandmother has received any middle school education
Household Asset Index	Index for durable goods (assets) owned by the household
Family Receives Minimum Living Standard Guarantee Payments (1 = Yes, 0 = No)	Whether family receives social security support from the government (a measure of poverty)
Panel C. Feeding Behaviors	
Exclusive Breastfeeding	Child was fed only through breastfeeding
Non-exclusive Breastfeeding, without Formula	Child was fed through breastfeeding and food (no formula)
Non-exclusive Breastfeeding, with Formula and Food	Child was fed through breastfeeding, food, and formula
Formula with Food	Child was fed through food and formula (no breastfeeding)
Food Alone	Child was fed only through food
Dietary diversity	Number of different food types child had consumed the previous day (according to WHO food categories)
Panel D. Health Outcomes	
Child had a fever in the past month (1 = Yes, 0 = No)	Caregiver recalls child having a fever in the month prior to survey administration
Child had diarrhea in the past month (1 = Yes, 0 = No)	Caregiver recalls child having diarrhea in the month prior to survey administration
Child had a cold in the past month (1 = Yes, 0 = No)	Caregiver recalls child having a cold in the month prior to survey administration
Child had a cough in the past month (1 = Yes, 0 = No)	Caregiver recalls child having a cough in the month prior to survey administration

**Table A2:** Summary statistics of outcomes and covariates by attrition status.

		Full Sample	Attrited Sample	Non-Attrited Sample	Difference: (2)–(3)
		Mean	Mean	Mean	*p*-Value
		Standard Deviation	Standard Deviation	Standard Deviation	
		(1)	(2)	(3)	(4)
Health outcomes					
(1)	Child had a fever in the past month	0.30 (0.46)	0.30 (0.46)	0.30 (0.46)	0.994
(2)	Child had diarrhea in the past month	0.37 (0.48)	0.36 (0.48)	0.37 (0.48)	0.717
(3)	Child had a cold in the past month	0.57 (0.50)	0.56 (0.50)	0.57 (0.50)	0.717
(4)	Child had a cough in the past month	0.45 (0.50)	0.47 (0.50)	0.45 (0.50)	0.707
Characteristics of infant					
(5)	Breastfeeding	0.58 (0.49)	0.62 (0.49)	0.58 (0.49)	0.249
(6)	Dietary diversity	3.00 (1.20)	2.99 (1.06)	3.00 (1.20)	0.916

Notes: Data source is authors’ survey. Descriptive statistics of child and household characteristics when children are 6–12 months of age. The first column shows the mean and standard deviation of each characteristic for the full sample; column 2 shows the characteristics of sample children who attrited between baseline and endline; and column 3 show the characteristics of children in the sample after attrition.
